# Endometrial claudin-4 and leukemia inhibitory factor are associated with assisted reproduction outcome

**DOI:** 10.1186/1477-7827-7-30

**Published:** 2009-04-19

**Authors:** Paulo C Serafini, Ismael DCG Silva, Gary D Smith, Eduardo LA Motta, André M Rocha, Edmund C Baracat

**Affiliations:** 1 Department of Gynecology, Faculdade de Medicina, Universidade de São Paulo, São Paulo, Brazil; 2 Huntington Reproductive Medicine, São Paulo, Brazil; 3 Department of Gynecology, Universidade Federal de São Paulo, São Paulo, Brazil; 4 Department of Obstetrics and Gynecology, Urology, Physiology, and Reproductive Science Program, University of Michigan, Ann Arbor, USA

## Abstract

**Background:**

Claudin-4 (CLDN4) is one of several proteins that act as molecular mediators of embryo implantation. Recently, we examined immunolabeling of leukemia inhibitory factor (LIF) in the endometrial tissue of 52 IVF patients, and found that LIF staining intensity was strongly correlated with successful pregnancy initiation. In the same set of patients, we have now examined endometrial CLDN4 expression, to see how expression intensity may vary with LIF. We examined CLDN4 in the luteal phase of the menstrual cycle, immediately preceding IVF treatment. Our aim was to compare expression of LIF and CLDN4 in the luteal phase, and document these patterns as putative biomarkers for pregnancy.

**Methods:**

Endometrial tissue was collected from women undergoing IVF. Endometrial biopsies were obtained during the luteal phase preceding IVF, and were then used for tissue microarray (TMA) immunolabeling of CLDN4. Previously published LIF expression data were then combined with CLDN4 expression data, to determine CLDN4/LIF expression patterns. Associations between successful pregnancy after IVF and combined CLDN4/LIF expression patterns were evaluated.

**Results:**

Four patterns of immunolabeling were observed in the endometrial samples: 16% showed weak CLDN4 and strong LIF (CLDN4^-^/LIF^+^); 20% showed strong CLDN4 and strong LIF (LIF^+^/CLDN4^+^); 28% showed strong CLDN4 and weak LIF (CLDN4^+^/LIF^-^); and 36% showed weak CLDN4 and weak LIF (CLDN4^-^/LIF^-^). Successful implantation after IVF was associated with CLDN4^-^/LIF^+^(p = 0.003). Patients showing this endometrial CLDN4^-^/LIF^+ ^immunolabeling were also 6 times more likely to achieve pregnancy than patients with endometrial CLDN4^+^/LIF^- ^immunolabeling (p = 0.007).

**Conclusion:**

The combined immunolabeling expression of CLDN4^-^/LIF^+ ^in endometrial tissue is a potential biomarker for predicting successful pregnancy in IVF candidates.

## Background

Embryonic implantation takes place on the uterine inner surface after a series of molecular and cellular interactions between the embryo and endometrium. Shedding of the zona pellucida, the apposition of the embryo to the endometrium, and embryonic invasion into the endometrial glandular epithelium and stroma, are all governed by multiple factors and highly active mechanisms [1]. Several types of proteins act as mediators of implantation [2], such as intracellular signal transducers, growth and differentiation factors, receptors, and cell surface adhesion molecules [3]. Furthermore, these proteins are responsible for the differentiation of endometrium, which facilitates embryo adhesion and invasion, gates infiltrating natural killer cells, and maintains the intercellular milieu [1].

Claudin-4 (CLDN4), initially named *Clostridium perfringens *enterotoxin receptor (CPER), may have an important role in implantation [4-6]. CLDN4 is a tetraspan protein with 27 kDa and shorter amino and carboxy terminals [7]. The claudin family is composed of more than 20 claudins [8] that are present in tight junctions, where they form aqueous and ion-selective aqueous pores [9,10].

Claudin-4 mRNA and protein show different patterns of expression during different cycle stages. cDNA microarray analysis of the midluteal phase endometrial transcriptome revealed increased CLDN4 mRNA, compared to other luteal phase stages (4–6). However, immunohistochemical analysis of CLDN4 protein expression in the endometrium during the secretory phase was weak or negative [11]. To date, there are no reports on the functional roles of CLDN4 during the implantation process, for either spontaneous or *in vitro *fertilization (IVF) pregnancies.

Recently, we examined luteal phase immunolabeling of endometrial leukemia inhibitory factor (LIF) prior to IVF treatment [12]. We found that strong endometrial LIF immunolabeling was associated with pregnancy. In fact, women expressing strong endometrial LIF immunolabeling were 6.4 times more likely to initiate a pregnancy than those with weak or absent LIF immunolabeling. The expression pattern of this cytokine during the menstrual cycle [13,14], and previous clinical associations between LIF deficiency and infertility [15], suggest LIF has a possible role in the implantation phenomenon.

The primary goal of the current study was to evaluate endometrial CLDN4 expression as a biomarker for pregnancy. Thus, we focused on endometrial CLDN4 immunolabeling in the luteal phase of the menstrual cycle, immediately preceding IVF treatment. In addition, we examined the association between endometrial CLDN4 and LIF immunolabeling.

## Methods

### Patients

The patients followed in this study were infertile women undergoing IVF treatments between July 2004 and August 2006. Briefly, all subjects were screened and assessed according to ASRM guidelines, with a detailed medical history, physical exam, and laboratory workup [16,17]. All participants had indications for IVF, and were counseled regarding the nature and purpose of the study. Approval for this research was obtained from both the Ethics Committee of the Department of Gynecology, and the Institutional Review Board of the Faculdade de Medicina da Universidade de São Paulo. All patients signed an informed consent form.

Eligibility criteria for inclusion in the study were: 1) presence of two functional ovaries; 2) an anatomically normal uterine cavity on the basis of recent hysterosalpingogram or hysteroscopy (≤ 6 months); 3) history of ≤ 3 attempts at IVF/ICSI; 4) early follicular phase (day 2 or 3) serum Follicle Stimulating Hormone (FSH) levels of ≤ 15 IU/L; 5) estradiol (E2) levels that were ≤ 60 pg/mL; 6) ho history of negative or low ovarian response in previous IVF/ICSI treatment; 7) body mass index (BMI) ≤ 28 kg/m^2^; 8) no treated endocrinologic disease; and 9) no gonadotropin therapy for the prior 3 months.

### Endometrial biopsy tissue processing

As previously described, endometrial biopsies were performed in an outpatient facility [12]. Briefly, after confirmation of ovulation by ultrasound examination at mid cycle, and when day 21 serum progesterone concentration was ≥ 3 ng/dL, endometrial biopsies were collected with a Pipelle endometrial suction curette (Pipelle de Cornier, Cooper Surgical, USA). Each patient was submitted to one biopsy collected during one of the three phases of the menstrual cycle: early (day 16–19), middle (day 20–24) and late (day 25–28), as well as immediately prior to commencement of GnRHa therapy. Endometrial tissue samples were fixed in formalin, then embedded in paraffin. Tissue blocks were sectioned at 3 – 4 μm, then processed for: 1) endometrial dating according to Noyes criteria [18], and 2) immunohistochemical labeling of CLDN4, described below.

### Tissue microarray and immunohistochemical analysis

To perform TMA analysis, 1 mm endometrial tissue samples were obtained from paraffin donor blocks representing the three different luteal stages of the luteal phase. Samples were acquired with a TMArrayer™ punch MP10-1.0 mm (Pathology Devices INC., USA), and arranged in the TMArrayer™ recipient block of 15 × 10 cores. Two cores of human prostate tissue were also included in the TMArrayer™ as positive controls for CLDN4 expression. All endometrial tissue samples were sectioned at 3–4 μm, then mounted on glass slides for CLDN4 immunohistochemistry [12].

Immunohistochemical labeling for CLDN4 was performed on 3 sections from each sample. For antigen retrieval, slides were incubated in citric acid solution (10 mM, pH 6.0) and processed in a microwave (1300 watts) for nine minutes [12]. Slides were washed in distilled water, and blocked in phosphate buffered saline (PBS) with blocking serum (10%). Slides were then incubated in primary antibody, a 1:200 dilution of rabbit polyclonal anti-CLDN4 (C-18: sc-17664, Santa Cruz Biotechnology INC., USA) in PBS supplemented with 2% of blocking serum for one hour at room temperature. Slides were then rinsed with PBS for 5 minutes, and incubated in a 1:200 dilution of goat anti-rabbit secondary antibody, linked to a streptavidin-biotin-peroxidase complex (StreptABComplex, DAKO, Denmark). The peroxidase was then exposed to a solution of diaminobenzidine (DAB, Sigma, EUA) in PBS, and allowed to react for 5 minutes, until the formation of a brown reaction product. Slides were then rinsed in PBS, and counterstained with Harris Hematoxylin (Merck, USA). After dehydration in a series of ethanols to xylene, slides were coverslipped in Histomount medium.

For all samples, CLDN4 immunolabeling was compared to positive prostate tissue controls by two experienced pathologists blinded to the sample identity and the purpose of the study. CLDN4 immunolabeling was scored according to intensity and frequency, following the method of Soini, with slight modifications [19]. The CLDN4 immunolabel score was computed as a multiplication of frequency × intensity, with results ranging between 0 and 12. Four categories were designated within this range of scores: negative (0 to 1.9); weak, (2.0 to 4.9); mild, (5.0 to 8.9); and strong (9.0 to 12.0). These results were then compared with endometrial LIF immunolabeling data from the same study group, which were previously published [12].

### Ovulation stimulation and IVF

The procedures for ovulation stimulation and IVF-ET have also been previously described in detail [12]. Briefly, patients were given daily subcutaneous injections of Leuprolide acetate (Lupron^®^, TAP Pharmaceuticals Products Inc.), during the mid- and late luteal phases of the previous menstrual cycle, to cause pituitary desensitization. Recombinant hFSH (Gonal-F^®^, Serono Laboratories) was then administered, in doses ranging from 150–300 UI, depending on patient age. The administration of the GnRH agonist was extended until the final day of follicle maturation, when at least 2 codominat follicles reach 18 mm. Subsequently, a maturational dose of recombinant hCG (Ovidrel^®^, Serono Laboratories) was injected to accommodate oocyte retrieval within 35–36 hours. Oocyte harvesting was carried out in an outpatient facility, under mild sedation and analgesia.

Following harvesting, oocyte fertilization procedures were initiated. Oocyte identification, gamete preparation and handling, insemination by ICSI, and embryo transfer, have all been previously described [20]. Embryo scoring was based on developmental stage and morphology, using established criteria. Average cumulative embryo scoring per transfer was calculated as the sum of transferred embryo scores/the total number transferred. All embryo transfers were carried out on day 3. Luteal phase hormonal support consisted of 1200 mg daily doses of micronized progesterone, beginning on the day after oocyte retrieval. A clinical pregnancy was defined by a pelvic ultrasound that showed a gestational sac with an embryonic heart activity. Birth rate in the study population was defined as the total successful births of a live child, divided by IVF treatment.

### Data analysis

A variety of statistical analyses were carried out to evaluate associations between immunolabeling patterns and pregnancy. Statistical association between CLDN4 immunolabel score and pregnancy was assessed by Chi-square analysis. To calculate an odds ratio for pregnancy, we used a binary logistic regression. In addition, previously published endometrial LIF immunolabeling data from the same group of patients [12] were combined with CLDN4 immunolabeling data, to determine coincident CLDN4/LIF expression patterns. Weak or mild immunolabeling was considered negative (^-^), and strong immunolabeling was considered positive (^+^). With this notation, combined immunolabeling patterns were classified into four categories: 1) strong CLDN4 and strong LIF (LIF^+^/CLDN4^+^); 2) strong CLDN4 and weak LIF (CLDN4^+^/LIF^-^); 3) weak CLDN4 and strong LIF (CLDN4^-^/LIF^+^); 4) weak CLDN4 and weak LIF (CLDN4^-^/LIF^-^).

Associations between successful pregnancy after IVF and each immunolabeling category were evaluated with Chi-square or Fisher's exact test. These tests were also used to determine association of immunolabeling patterns with either histological stage of the luteal phase, or infertility cause (endometriosis, polycystic ovary syndrome, tubal patency failure and male infertility).

Statistically significant differences in the proportion of pregnancy/non-pregnancy, and birth/non-birth among the CLDN4/LIF immunolabeling categories were examined by a Z-test for two proportions. Frequencies of pregnancy and birth were tested for correlation by calculating a Pearson's correlation coefficient (r).

In addition, general IVF parameters were tested for correlation with immunolabeling patterns. First, women's age, total rFSH dose, number of transferred embryos, number of top quality embryos, and mean score per embryo were submitted to a Kolgomov-Smirnov test, to assess normal distribution, then an F-test, to assess data homogeneity. Differences in immunolabeling patterns among pregnant and non-pregnant women were compared by Student's t test. These data among different immunolabeling categories were further compared by ANOVA and Tukey's test. All data are expressed as mean ± standard deviation (SD), and significance level was set at p < 0.05.

## Results

### CDLN4 immunolabel in stages of luteal phase

**Figure 1:**
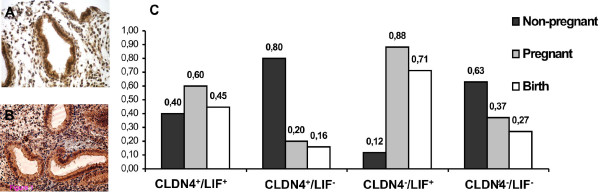
**Examples of strong immunolabeling of A) CLDN4 and B) LIF**. C) The frequency of pregnancy, non-pregnancy, and birth, in patients from each of the four CLDN4/LIF endometrial immunolabeling categories.

A total of 52 endometrial biopsy core samples were obtained from the 52 women in the study group (mean age, 35 ± 5 years). Across all phases, endometrial CLDN4 immunolabeling was mild in 16%, strong (Figure 1A) in 48% and absent in 36%.

All endometrial tissue samples were histologically defined as either early luteal phase (n = 23), midluteal phase (n = 18), or late luteal phase (n = 11). Among early luteal phase samples, CLDN4 immunolabeling was absent in 50% (n = 11) of patients, mild in 26% (n = 6), and strong in 26% (n = 6). Among midluteal phase samples, CLDN4 immunolabeling was absent in 17% (n = 3) of patients, mild in 11% (n = 2), and strong in 72% (n = 13). Among late luteal phase samples, CLDN4 immunolabeling was absent in 40% (n = 4), and strong in 60% (n = 7) of patients. There was no association between CLDN4 expression and stage of luteal phase.

### CDLN4 and infertility

Among the 52 patients, causes of infertility were: endometriosis (27%, n = 14), polycystic ovary syndrome (25%, n = 13), tubal patency failure (13%, n = 7) and male infertility (35%, n = 18). Infertility cause was not associated with CLDN4 immunolabeling (p = 0.09) (Table 1); and, neither with women who did not establish pregnancy (p = 0.8). However, strong CLDN4 immunolabeling was associated with failure to establish pregnancy after IVF (p = 0.01). These patients were 10.5 times less likely to achieve pregnancy than those with weak immunolabeling.

**Table 1:** Distribution of infertility etiology identified among patients in the study among the CLDN4/LIF immunolabeling categories

CLDN4/LIF categories	Infertility cause			
	Male factor	Tubal factor	Polycystic ovary syndrome	Endometriosis
CLDN4^+^/LIF^+^	3	2	1	3
CLDN4^-^/LIF^+^	4	9	9	6
CLDN4^+^/LIF^-^	7	2	1	4
CLDN4^-^/LIF^-^	4	9	9	6

### A review of LIF immunolabeling in stages of luteal phase

CLDN4 immunolabeling was then compared to previously reported LIF immunolabeling. To review, LIF immunolabel was weak in 5.8% (n = 3), mild in 61.5% (n = 32) and strong (Figure 1B) in 32.7% (n = 17). Among early luteal phase samples, 35% (n = 8) showed strong LIF immunolabeling, while 60% (n = 14) showed mild, and 5% showed weak (n = 1). Of the midluteal samples, 50% (n = 9) showed strong, 44% (n = 8) showed mild, and 6% (n = 1) showed weak LIF immnolabeling (Figure 1B). Of the late luteal phase samples, 82% (n = 9) showed mild, 18% (n = 2) showed strong, and none showed weak LIF immunolabeling. There was no association between LIF immunolabeling and stage of luteal phase. However, LIF expression was associated with pregnancy, and patients expressing strong LIF immunolabel in the endometrium were 6.4 times likely to achieve pregnancy than those with weak or mild staining [12].

### Comparison of CLDN4 and LIF within each patient

We then compared the CLDN4 and LIF immunolabeling data within each patient. Strong immunolabeling for LIF was not associated with strong immunolabeling for CLDN4 (Table 2). Four patterns of immunolabeling were observed in the endometrial samples: 16% showed weak CLDN4 and strong LIF (CLDN4^-^/LIF^+^); 20% showed strong CLDN4 and strong LIF (CLDN4^+^/LIF^+^); 28% showed strong CLDN4 and weak LIF (CLDN4^+^/LIF^-^); and 36% showed weak CLDN4 and weak LIF (CLDN4^-^/LIF^-^).

**Table 2:** Distribution of the number of patients among endometrial CLDN4/LIF immunolabeling intensities

**Endometrial expression**	**LIF^+^**	**LIF^-^**
**CLDN4^+^**	9	14
**CLDN4^-^**	7	22
Chi-square: 1.353	p = 0.245	

Chi-square for association between positive and negative immunolabelings for CLDN4 and LIF

### CLDN4/LIF category and patient parameters

Immunolabeling patterns were then compared with parameters of the patient population. There were no associations between endometrial CLDN4/LIF immunolabeling category and cause of infertility (Table 2; p = 0.165), or stage of the luteal phase (p = 0.09). The overall pregnancy rate in the study group was 39% (n = 20).

Interestingly, there was an association between CLDN4^-^/LIF^+ ^immunolabeling category (p = 0.01) and pregnancy. The frequency of pregnancy (fp) was higher than the frequency of non pregnancy (fnp) in patients whose samples showed endometrial CLDN4^-^/LIF^+ ^immunolabeling (fp = 0.88; p = 0.035; 95% CI = 0.47; 0.99), and lower than fnp in those with CLDN4^+^/LIF^- ^immunolabeling (fp = 0.2; p = 0.013; 95% CI = 0.01; 0.40). The proportion of pregnant and non-pregnant patients was similar between women with endometrial CLDN4^+^/LIF^+^immunolabeling (fp = 0.6; p = 0.754; 95% CI = 0.26; 0.87) and CLDN4^-^/LIF^- ^immunolabeling (fp = 0.63; p = 0.359; 95% CI = 0.38; 0.83) (Figure 1c). Strikingly, patients with CLDN4^-^/LIF^+ ^endometrial immunolabeling were 36 times more likely to get pregnant, compared to those with CLDN4^+^/LIF^- ^immunolabeling (p = 0.007; 95% CI = 2.69, 481.23). In addition, these CLDN4^-^/LIF^+ ^patients were 10.29 times more likely to achieve pregnancy than those with CLDN4^-^/LIF^-^immunolabeling (p = 0.048; 95% CI = 1.02, 103.95). Though these CLDN4^-^/LIF^+ ^endometrial immunolabeling patients were also 4 times more likely to achieve pregnancy than those with CLDN4^+^/LIF^+^immunolabeling, this odds ratio was not significant (p = 0.27; 95% CI = 0.34, 47.11).

At the opposite extreme, patients exhibiting CLDN4^+^/LIF^-^endometrial immunolabeling showed much less pregnancy success. These patients had 0.89 less chance of pregnancy than those with CLDN4^+^/LIF^+ ^immunolabeling (p = 0.028; 95% CI = 0.02, 0.79). Furthermore, patients exhibiting CLDN4^+^/LIF^- ^endometrial immunolabeling showed 0.71 less chance of pregnancy compared to those with CLDN4^-^/LIF^- ^immunolabeling, but this odds ratio was not significant (p = 0.16; 95% CI = 0.05, 1.67).

And finally, the absence of immunolabel for either CLDN4 or LIF (CLDN4^-^/LIF^-^) did not show a different chance of pregnancy than those with strong immunolabel for both proteins (vs. CLDN4^+^/LIF^+^, (p = 0.23; odds ratio = 0.39; 95% CI = 0.08, 1.87). Data is summarized in table 3.

**Table 3:** Odds ratio(OR) * for pregnancy, 95% confidence interval (95%CI) and p-value (p) for CLDN4/LIF categories.

	**CLDN4^+^/LIF^+^**	**CLDN4^+^/LIF^-^**	**CLDN4^-^/LIF^-^**
**CLDN4^-^/LIF^+^** (OR; 95%CI; p)	4; 0.34–47.11; 0.27	36; 2.69–481.2; 0.007	10.29; 1.02–103.95; 0.048
**CLDN4^+^/LIF^-^** (OR; 95%CI; p)	0.89; 0.2–0.79; 0.028	0.71; 0.05–1.67; 0.16	
**CLDN4^-^/LIF^-^** (OR; 95%CI; p)	0.39; 0.08–1.87; 0.23		

*Odds ratio were calculated for each category in the lines in relation to the each category in the columns.

### Birth rates and CLDN4/LIF immunolabeling category

The frequency of birth (fb) was correlated to fp in the study group (r = 0.997; p = 0.003), so associations with CLDN4/LIF endometrial immunolabeling followed a pattern similar to those seen with fp. The fb was lower than the frequency of non-birth (fnb) in patients with CLDN4^+^/LIF^- ^endometrial immunolabeling (fb = 0.16; p = 0.0001; 95% CI = -0.95; -0.45) and CLDN4^-^/LIF^- ^immunolabeling (fb = 0.27; p = 0.0001; 95% CI = -0.71; -0.19). Also, the proportion of births was similar in women with endometrial CLDN4^-^/LIF^+ ^immunolabeling (fb = 0.71; p = 0.076; 95% CI = -0.04; 0.9), and CLDN4^+^/LIF^+^immunolabeling (fb = 0.45; p = 0.635; 95% CI = -0.34; 0.57) (Figure 1C). Odds ratio for birth is summarized in table 4.

**Table 4:** Odds ratio(OR)* for birth, 95% confidence interval (95% CI) and p-value (p) for CLDN4/LIF categories.

	**CLDN4^-^/LIF^-^**	**CLDN4^-^/LIF^+^**	**CLDN4^+^/LIF^-^**
**CLDN4^+^/LIF^+^** (OR; 95%CI; p)	2.13; 0.42–10.73; 0.358	0.32; 0.04–2.62; 0.288	4.8; 0.65–35.2; 0.123
**CLDN4^+^/LIF^-^** (OR; 95%CI; p)	0.44; 0.08–2.6; 0.368	0.07; 0.01–0.61; 0.017	
**CLDN4^-^/LIF^+^** (OR; 95%CI; p)	6.67; 1.01–44.10; 0.049		

*Odds ratio were calculated for each category in the lines in relation to the each category in the columns.

### Overall patient statistics for IVF

We calculated mean IVF characteristics for the study group. On average, patients experienced 1.51 ± 1.22 previous IVF cycles before consenting to this study. Ovarian stimulation was accomplished with 2605.00 ± 995.00 UI of rFSH, and an average of 3 ± 1 embryos were transferred. Of the embryos transferred, 1 ± 1 was classified as top quality, and the cumulative score/embryos transferred/procedure was 20 ± 8.

We also compared characteristics of pregnant and non-pregnant patients. Pregnant patients were typically younger than non-pregnant patients (Table 2), and the increase of one year in maternal age accounted for a 30% decrease in pregnancy chances (p = 0.002). Similar among pregnant and non-pregnant patients were: number of previous IVF cycles, rFSH dose required for Controlled Ovarian Stimulation (COS), number of transferred embryos, number of top quality transferred embryos, and the mean embryo score (Table 5). These parameters were also similar among all CLDN4/LIF immunolabel categories (Table 6).

**Table 5:** IVF parameters in the study group

	**Pregnant** **(mean ± SD)**	**Non-pregnant** **(mean ± SD)**
**Maternal age (yrs)**	33.1 ± 4.17^a^	36.06 ± 4.7^b^
**Number of previous IVF cycles**	1.32 ± 1.36^a^	1.66 ± 1.12^a^
**rFSH total dose (IU)**	2476 ± 999^a^	2679 ± 1,000^a^
**# transferred embryos**	3.2 ± 0.8^a^	2.9 ± 0.9^a^
**# top quality embryos**	1 ± 1.2^a^	0.55 ± 0.9^a^
**Mean score per embryo**	22.8 ± 7.5^a^	18.7 ± 8.2^a^

Different letter indicate p < 0.05

rFSH total dose = amount used to accomplish ovulation induction

**Table 6:** IVF parameters separated into CLDN4/LIF immunolabeling categories

	**CLDN4^+^/LIF^+^** **(mean ± SD)**	**CLDN4^+^/LIF^-^** **(mean ± SD)**	**CLDN4^-^/LIF^-^** **(mean ± SD)**	**CLDN4^-^/LIF^+^** **(mean ± SD)**
**Maternal age (yrs)**	36.5 ± 3.2	35.4 ± 4	35 ± 4	35.4 ± 5.5
**# of previous IVF cycles**	1.18 ± 1.04	1.65 ± 1.32	1.34 ± 1.45	1.55 ± 1.63
**rFSH total dose (IU)**	2340 ± 708	2609 ± 1,151	2697 ± 1028	2721 ± 1067
**# of transferred embryos**	3.2 ± 0.8	3 ± 0.7	3.1 ± 1	2.5 ± 0.8
**# of top quality embryos**	0.9 ± 0.7	0.7 ± 0.3	0.8 ± 1	0.2 ± 0.3
**Mean score per embryo**	21.6 ± 6.8	15.2 ± 6.9	18.2 ± 6.9	20.5 ± 8.3

## Discussion

Our observations of endometrial CLDN4 and LIF immunolabeling during the luteal phase in IVF patients revealed a specific expression profile associated with successful pregnancy. While the role of CLDN4 in the optimization of endometrium for implantation is not clear, our results suggest that higher levels of CLDN4 are associated with lower pregnancy and birth rates. In addition, our CLDN4 immunolabeling data are agreement with previous cDNA microarray studies, wherein the frequency of strong immunolabeling for CLDN4 peaked in the midluteal phase, and remained high during the late luteal phase [2].

The influence of CLDN4 may be related to its presence in tight junctions. The traffic of small solutes between different intercellular compartments is controlled by pores in tight junctions [21]. Indeed, a crucial intercellular compartment is the microenvironment between the differentiating endometrium and the trophoblast. Pore selectivity is determined by its protein composition, and CLDN4 is one of the component proteins [9,21]. Specifically, pores containing CLDN4 are selectively permeable to chloride ions, and exclusive of sodium ions [22].

An increase of CLDN4 mRNA expression during the implantation window has been reported by several groups, and these findings support a functional role for this tight junction protein in implantation [4-6]. At odds with these reports, we observed that strong immunolabeling of CLDN4 was associated with non-pregnancy. Another study reported only weak endometrial CLDN4 expression in normal women during the secretory phase [11]. A possible mechanism supporting the association of high CLDN4 expression with non-pregnancy could be an adverse concentration of chloride caused by an increase in CLDN4-containing pores. Under these circumstances, tight junctions may disadvantageously bias the sodium/chloride regulation of the stromal paracellular milieu governed by CLDN4 pores [22].

Two studies proposed that CLDN4 and LIF are upregulated by the action of progesterone [23,24]. However, our four categories of CLDN4/LIF immunolabeling demonstrate no association between the immunolabeling intensity of these endometrial proteins. The apparent independent expression of CLDN4 and LIF suggests that they may be controlled by different pathways, triggered by the balanced relationship between progesterone receptors A and B [3] Though the expression of CLDN4 and LIF appear unrelated, a survey of independent pathways related to the implantation phenomenon might be advantageous for the assessment of IVF success.

In the current study, the combined assessment of endometrial CLDN4 and LIF immunolabeling during the luteal phase preceding an IVF treatment revealed that certain expression patterns can be predictive of pregnancy before IVF treatment. These data also re-emphasize the importance of LIF, as LIF^+ ^immunolabeling is strongly associated with a higher chance of pregnancy, an outcome that is not affected by strong expression of CLDN4.

While the association of strong CLDN4 expression with non-pregnancy could be related to changes in ion regulation dynamics, the presence of strong LIF immunolabel appears to reduce this association. Our data show that the association of strong CLDN4 immunolabeling with non-pregnancy is weakened by the strong expression of LIF immunolabeling. Specifically, the detrimental effect of strong CLDN4 immunolabeling is not observed in CLDN4^+^/LIF^+ ^patients. Rather, the more potent contributor to the success or failure of implantation or pregnancy appears to be the absence of LIF, though CLDN4 does appear to contribute in part.

In this study group of IVF patients, birth rates were highly correlated to pregnancy rates. The frequency of births was lower than the frequency of non-birth in patients with CLDN4^+^/LIF^- ^and CLDN4^+^/LIF^+ ^immunolabel categories, yet similar among the CLDN4^-^/LIF^- ^category. Furthermore, these frequencies of pregnancy and birth tended to be higher in patients in the CLDN4^-^/LIF^+ ^immunolabel category. These patterns of birth frequency reflect the correlation of pregnancy rates, and thus may not directly implicate endometrial CLDN4/LIF patterns in the processes following implantation.

These results raise the question, "What are the clinical/therapeutic implications of a CLDN4^+^/LIF^- ^profile, or other less favorable endometrial characterizations?" Indeed, patients undergoing infertility workup and possible Assisted Reproduction Treatment programs should be informed of their reproductive prognosis and chances of success or counseling. The endometrial characterization described here may help shape expectations for these patients. Thus, the development of subsidiary exams to assess the endometrium alongside other factors affecting implantation may invigorate the counseling of infertile couples.

The therapeutic value of endometrial characterization is not as easily defined. Our data suggest that compensation for low LIF expression may increase pregnancy chances, and possibly counteract equally unfavorable high levels of endometrial CLDN4. However, controlled increase of endometrial LIF is not yet clinically possible. The current understanding that embryonically-derived hCG upregulates expression of endometrial LIF [25], and that local instillation of hCG increases LIF expression [26], supports a therapeutic approach of worthy of further investigation.

The pattern of steroid concentration varies with age. In both natural and stimulated cycles of younger women, the "endometrial proteome profile" might not be similar to those in older women, as shown for patients that did not achieve pregnancy [21,27]. Due to ethical limitations, biopsies of endometrial tissue from failed pregnancies are not easily available, thus limiting a direct evaluation of CLDN4/LIF immunolabeling in these cases. There are further limitations on examination at the earlier timepoint of embryo transfer. Nevertheless, a CLDN4/LIF endometrial profile gathered from endometrial biopsies obtained during the implantation window of a spontaneous cycle could generate a useful criteria for an endometrium that is optimal for implantation. Clinical comparison to endometrial biopsies following ovarian stimulation would help forecast pregnancy success after an IVF treatment.

As observations from clinical research can often provide valuable feedback for basic research, our data may help researchers who optimize Assisted Reproduction Technology, or those working in basic areas in Reproductive Biology. We report these data with the hope that they will also support the generation of new research questions and therapeutic innovations, in the sprit of Translational Medicine.

## Conclusion

Determination of the relative endometrial CLDN4/LIF expression in a spontaneous menstrual cycle preceding an IVF treatment can be used as a biomarker of endometrial receptivity.

## Competing interests

The authors declare that they have no competing interests.

## Authors' contributions

Each author's contribution to the work was as follows. PCS performed patient selection, endometrial biopsies, TMArray reading, data analysis and manuscript drafting. IDCGS contributed to the study design, protein selection and immunohistochemistry. GDS contributed to protein selection and manuscript drafting. ELAM performed patient selection and endometrial biopsies, and contributed to manuscript drafting. AMR contributed to statistical analysis and manuscript drafting. ECB contributed to protein selection and manuscript drafting. All authors read and approved the final manuscript.
